# Development of personalized machine learning models for predicting panic attacks in college students

**DOI:** 10.3389/fdgth.2026.1978650

**Published:** 2026-10-30

**Authors:** Dheeraj Dhanvee Kairamkonda, Josh Cherian, Guido Mascia, Johanna E. Hidalgo, Julia N. Kim, Jordan Llorin, Kathryn Stanton, Jennifer Ha, Peter Sheridan Dodds, Matthew Price, Christopher M. Danforth, Joseph Sartini, Ellen W. McGinnis, Ryan S. McGinnis

**Affiliations:** 1 Center for Remote Health Monitoring, Wake Forest University School of Medicine, Winston-Salem, NC, United States; 2 Team LEMURS, University of Vermont, Burlington, VT, United States

**Keywords:** digital biomarkers, digital phenotyping, mental health, panic attack, personalized models

## Abstract

Panic attacks (PAs) are unexpected episodes of intense fear and anxiety, accompanied by cognitive and physical symptoms that significantly impair day-to-day functioning. This study proposes a machine learning approach to predict PA risk in the upcoming week in individuals across mental health conditions, enabling timely and potentially preventive mental health intervention. We evaluate the approach in a longitudinal sample of behavioral and physiological data collected using Oura rings from 39 college students over six months. We assessed multiple prediction algorithms applied within both personalized and generalized frameworks, fitting unique models for each participant and pooling data, respectively. Across all 39 participants, personalized models using logistic regression achieved a pooled PR AUC lift of 1.17 (95% CI: 1.05–1.25) over chance-level prediction (*p* = .035), whereas the generalized model did not differ from chance (PR AUC lift of 0.99; 95% CI: 0.90–1.08); in a direct comparison, personalized models outperformed the generalized model (lift ratio=1.18, *p* = .047). Performance varied across participants: when personalized models were grouped by unsupervised clustering of test-set PR AUC lift, models clustered as well-fit (*n* = 8) showed a pooled PR AUC lift of 2.26 (95% CI: 1.50–2.89) and models clustered as poor-fit (*n* = 31) a lift of 1.09 (95% CI: 0.98–1.17). Because these groups were defined by test-set performance, they describe observed heterogeneity and require validation in independent data. Together, our findings point towards the potential value of personalized modeling approaches and suggest that passively collected daily measurements of sleep, activity, and physiology may carry signal for predicting PAs, which could ultimately be leveraged to identify individually tailored lifestyle interventions to reduce PA risk.

## Introduction

Panic attacks (PAs) are sudden and intense episodes of fear or anxiety accompanied by a range of physical and cognitive symptoms such as chest pain, tachycardia, hyperventilation, sweating, nausea, dizziness, derealization, and fear of losing control or dying (1). More than one in ten people in the US experience a PA each year (2), and those PAs are associated with poorer overall well-being (3), more missed work (4), more visits to the emergency department (3, 5), and suicide (6).

PAs are transdiagnostic, and yet, prior work on PAs is almost exclusively within panic disorder (PD). Research on the mechanisms and treatment of PAs has focused almost exclusively on the individuals who experience PAs with PD (7). While this work has generated substantial neurobiological and physiological insights into PD, a condition affecting approximately 2.7% of U.S. adults annually, it leaves the much larger population (∼8% of U.S. adults) of individuals with recurrent PAs but no PD (7) uncharacterized. The DSM-5 reclassified PAs as a transdiagnostic specifier that can be applied across conditions, reflecting their occurrence across a wide range of disorders (8, 9) and association with greater illness severity, impairment, and service utilization (7, 10, 11). For instance, PAs occurring within depression predict future suicidal ideation and attempts (11, 12). Together, these findings position PAs as a clinically meaningful, cross-cutting risk marker rather than a disorder-specific phenomenon.

To augment clinician-in-the-loop treatments for PAs, particularly in the context of limited access to mental health professionals, there is growing interest in scalable interventions that leverage continuous self-monitoring and self-management (13, 14). Such approaches align with patient-reported needs for timely support that can anticipate and help prevent imminent PAs (15–17), addressing critical gaps in care during periods of heightened risk when clinician-delivered treatment is often unavailable. Just-in-time adaptive interventions (JITAIs) offer a promising framework for extending evidence-based care beyond the clinic; however, their clinical utility depends on accurate, personalized prediction of PAs (18). Developing such systems requires predictive models that identify not only salient risk features but also their temporal dynamics leading up to an attack (19). Reliable early warning could enable timely delivery of evidence-based strategies or reinforcement of skills learned in psychotherapy, thereby increasing the reach, responsiveness, and scalability of PA interventions.

Building on this motivation, several works, including our own, have explored predictive modeling approaches for forecasting PAs, with promising results that have begun to identify candidate features and their temporal patterns. Promising signals have been identified using passively collected physiological and behavioral data from wearable sensors (20–25), supported by complementary laboratory studies. These include features related to inactivity and exercise intensity, sleep characteristics such as wake-up regularity, respiratory features including breathing rate, and cardiovascular measures such as heart rate and heart rate variability (26–30). In parallel, self-reported stress and mental health symptoms have also demonstrated predictive value (16, 31–35). However, much of the existing work has focused exclusively on individuals with panic disorder (20, 36), who make up only about 17% of individuals who suffer from PAs (2). Work from Tsai et al. reported AUCs of 0.87 (20) and 0.99 (36) for predicting PAs in samples exclusively diagnosed with panic disorder. In both studies, performance was found to be driven primarily by self-reported questionnaires rather than objective wearable data, and there was no ablation analysis conducted to isolate the impact of wearable data on performance.

Our prior work suggests that the presence and type of psychopathology meaningfully influences which features are most informative (19), highlighting limitations of one-size-fits-all models. Furthermore, since the behavioral and physiological responses to stressors differ significantly across mental health diagnoses (37–42) and even individuals (43–46), this highlights the need for personalized predictive models that can capture this heterogeneity and individual-specific variation in predicting PAs, thereby supporting clinically effective and scalable JITAIs. Panic disorder and panic attacks are not evenly distributed across sociodemographic groups: panic disorder is roughly 2.5 times more prevalent in women than men (47), and Black and Hispanic patients are diagnosed and treated for anxiety-related conditions at lower rates than White patients in primary care settings (48), even when reported symptoms are comparable. Our sample reflects this pattern in part; the cohort was predominantly women and White- which should be considered when interpreting the generalizability of the resulting models.

In this work, we aim to use ML approaches to develop models and internally validate them for predicting PAs using behavioral and physiological measures from the Oura ring in a sample of people with a variety of mental health conditions who experience PAs (Figure 1). To our knowledge, this is the first paper to propose personalized models for predicting panic attacks. In this effort, we explore generalized models that work across participants as well as models personalized for each individual. We further interrogate model performance by PA frequency and observe two case studies highlighting potential lifestyle modifications that could reduce individual PA risk. This model development serves as an exploration of the potential of personalized models in effectively predicting panic attacks, which can be further tested externally on diverse populations or larger samples. These algorithms may allow development of intervention tools for people suffering from PAs to provide timely intervention even before they experience a PA.

**Figure 1:**
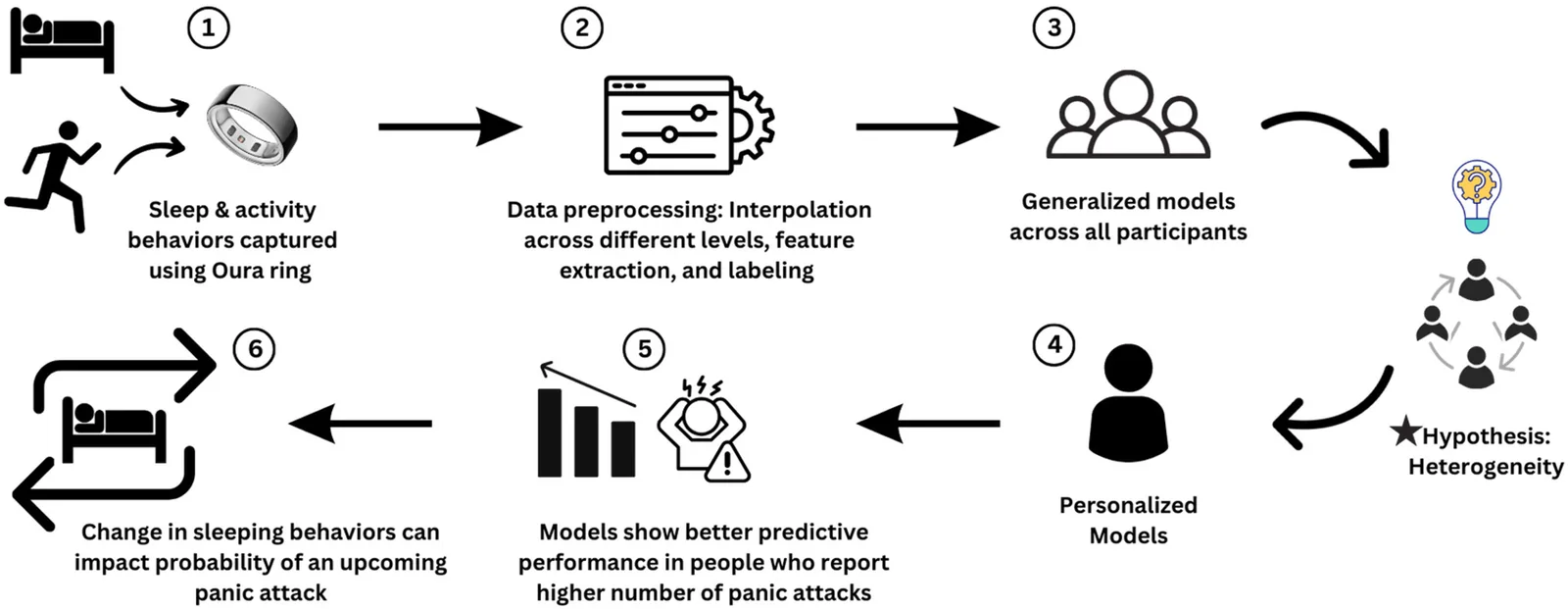
Overview of the flow of the paper, illustrating the progression from data collection to *post-hoc* analyses. (1) Behavioral data were collected using Oura rings, which provide daily summaries of sleep and activity. (2) The data were preprocessed using multiple interpolation strategies to fill gaps ranging from 0 to 7 consecutive days between observations. Features were extracted using 7-day sliding windows with a step size of 1 day, computing statistical measures such as the mean, standard deviation, and 5th and 95th percentiles for each feature. Each window was labeled as 0 or 1 based on whether a panic attack occurred in the week following the last day of the window. (3) Generalized models trained on the interpolated data did not demonstrate strong predictive performance, motivating the hypothesis (★) that predictors of panic attacks vary across individuals. (4) To test this hypothesis, personalized models were developed, which modestly outperformed the generalized model. (5) *post-hoc* exploratory analyses examined the level of heterogeneity in the predictors of panic attacks across participants and the influence of behaviors on upcoming panic attack risk. (6) Finally, analyses of behavioral correlates examined how changes in daily behaviors relate to changes in the probability of an upcoming panic attack.

## Methods

### Data collection

This is a secondary analysis of the data collected as part of the Lived Experience Measured Using Rings Study (LEMURS), where the primary aim was to study mental health and wellness behaviors in college students (49–52). This study was approved by the University of Vermont (UVM) Institutional Review Board, and all participants signed informed consent prior to data collection. Participants were all full-time first- or second-year college students at the University of Vermont (UVM). Eligible participants were between 18 and 24 years of age, full-time students at UVM, and owned an iPhone model 6S/SE or newer. Participants were asked to wear a provisioned Oura ring continuously throughout the academic year. Participants completed surveys about their emotional and behavioral health at the beginning of the study (baseline), biweekly throughout the year, and also daily during four 21-day ’sprints’ via a bespoke app (49). Participants were not involved in the design or conduct of this study.

### Measures

#### Oura data

The Oura ring captures both behavioral and physiological data continuously. Daily summary metrics (one value per variable) were derived as Oura metrics. The behavioral metrics comprise measures related to sleep and activity. Sleep measures encompass variables such as sleep onset latency, sleep staging, and sleep efficiency, among others, while the activity measures include time spent in different intensity activities (high, medium, low, inactivity), calories burned, and average metabolic equivalents (METs) (53). Physiological metrics include heart rate, heart rate variability, breathing rate, and body temperature recorded during sleep. The validity of the Oura Ring has been widely established in the literature through comparative studies with other commercially available wellness devices and gold-standard measurement technologies [e.g. (54–56),], as well as its use in studies addressing mental health and behavioral outcomes (57, 58).

#### Daily surveys

As part of the data collection process, daily surveys were deployed during the 2023-2024 academic year (230 days) during “sprints”. Sprints were established to concentrate participant burden into brief periods to reduce survey fatigue across the year. Participants were randomly divided into three groups and assigned to four 21-day sprints of daily surveys, followed by a 42-day gap with no daily surveys. Consequently, daily surveys occurred on the same calendar dates for some participants and on different calendar dates for others. This resulted in each participant completing 84 total days of daily surveys. On daily surveys, participants were asked to check a box if they had experienced a “panic attack” that day. There was no further definition of a panic attack, so endorsement relied on the participant's own understanding of the term.

#### Biweekly surveys

Biweekly surveys were completed in the same period, where the participants were asked to complete the Depression Anxiety Stress Scales (DASS) (59) every two weeks to quantify stress, anxiety, and depression levels (Range: 0–21).

#### Diagnostic interviews

Mental health diagnoses for each participant were determined using the Mini International Neuropsychiatric Interview (MINI) (60). MINIs were conducted in-person by trained research staff. Diagnostic groups used herein included those with a mental health diagnosis of Panic Disorder, Depression, Anxiety, or having no mental health diagnoses. Note that all three mental health diagnoses listed here are not mutually exclusive since participants have multiple diagnoses.

#### Dataset, data preprocessing, and feature extraction

Oura provides daily summary metrics, where each observation represents an aggregate of that day's sleep, activity, and physiological data. When the daily summary was available but the value for a specific variable was missing, such values were imputed using the participant-specific mean. For the days where all summary data were missing, linear interpolation was used to estimate the values, provided the gap did not exceed seven days; both uses data preceding the forecast periods.

The following statistical measures were extracted for each Oura-derived sleep, activity, and physiological metric from a 7-day sliding window with a step size of 1 day: mean, standard deviation, 5th and 95th percentile. This yielded a total of 148 features. Windows were considered valid if the days were consecutive and there was at least one panic report (yes or no) in the week following the window on which features were extracted. Windows were labeled 1 if at least one observed day in the subsequent week reported a panic attack, and 0 if none of the observed days reported a panic attack. The “Panic attack events reported” in Table 1 represents the panic events reported by the participants as a part of the daily survey while “Panic attack (post processing)” represents these windows that we built in the preprocessing. This setup is built to predict PA risk in the upcoming week. Features were standardized using participant-specific means and standard deviations only using the values from the training set of each participant. Across the 39 participants in the final analytic dataset, the ratio of non-PA to PA days, computed on each participant's full set of training and test observations combined, varied considerably (M = 2.12, SD = 1.30, range = 0.51–5.14), reflecting substantial between-participant variability in class balance.

**Table 1:** Participants characteristics and data summary.

Variable	Mean (SD)	*n* (%)
Data		
Sample size		39
Oura daily observations across participants	70 (14)	—
Train observation across participants	47 (15)	
Test observation across participants	24 (6)	
Panic attack events reported	6 (4)	—
Panic attack (post processing)	26 (11)	
Test prevalence (post processing)	0.45 (0.21)	
Stressors per participant	72 (42)	—
DASS Stress score	17 (7)	—
DASS Anxiety score	13 (7)	—
DASS Depression score	14 (10)	—
Mental Health Diagnosis		
Sample Size		32
Panic Disorder	—	43.8%
Anxiety Disorder	—	53.1%
Depression	—	78.1%
No Diagnosis	—	9.4%

Diagnostic categories are non-mutually exclusive; percentages are of the 32 participants with a documented diagnosis and sum to >100%. Diagnosis was not available for the remaining 7 of the 39 participants.

Since depression, anxiety, and stress scores (DASS) are reported biweekly, for each report, the corresponding scores were assigned to the 14 days preceding the report date. DASS scores reflect a retrospective summary collected at the end of these 14 days. Self-reported daily stressors were aggregated into a single feature representing the total number of stressors reported each day. Both the DASS scores and the aggregated stressor feature underwent the same preprocessing pipeline as the Oura-derived metrics, including a 7-day sliding window with a step size of one day, statistical feature extraction (mean, standard deviation, 5th and 95th percentiles), and participant-wise standardization.

#### Data exclusion criteria & participant characteristics

Of the 197 participants who reported at least one panic attack via daily surveys, 187 had sufficient Oura data to be included in the personalized modeling pipeline, of whom 148 were excluded from the analysis: 129 for having only one class (PA or non-PA) represented in their training set, test set, or both; 8 for having fewer than two sprints; 9 for having fewer than 7 training or 7 test observations after the chronological split; and 2 for having fewer than 10 total daily observations. Using these criteria, we were left with 39 participants. These criteria were necessary because PR AUC is undefined for a test set containing a single class, and a classifier cannot be trained on data lacking outcome variability; participants were therefore excluded when their data could not support a well-defined training or evaluation split, not based on any measure of model performance.

Thirty-nine participants who self-reported at least two panic attacks while wearing their Oura ring during the academic year were included in the current study. Diagnostic status was available for 32 of these 39 participants; among them, most had a mental health diagnosis (90.6%; see Table 1). Sex was available for 37 of 39 participants (2 missing); among them, 91.9% were women and 8.1% were men. Most participants were 18 years old (M = 18.6, SD = 0.6). Race/ethnicity was available for 37 of 39 participants (2 missing); because participants could select more than one category, percentages do not sum to 100%. Among these 37, 83.8% identified as White, 18.9% as Hispanic, 16.2% as European, 8.1% as Other Race, 5.4% as Middle Eastern, 5.4% as East Asian, and 2.7% each as African American and Southeast Asian.

#### Machine learning for predicting panic attacks

The machine learning approach we adapted can be categorized into two types. First, we built a generalized model across all modeled participants to identify common behavioral patterns preceding PAs. Second, we built personalized models for each participant individually. For both approaches, each participant's longitudinal data was first segmented into contiguous “sprints” of consecutive daily observations. Data from each participant's final sprint were reserved as the test set, and all preceding sprints were used for training; this respects the time-series correlations within sprints and avoids temporal leakage between train and test. This setup is designed to predict the risk of a panic attack in the upcoming week.

For the generalized model, training and test sets were pooled across all modeled participants using this same sprint-based split, restricted to the identical participants and rows used in the personalized comparison so the two approaches are evaluated on directly comparable data. For both the general and personalized models, four prediction algorithms were explored: Logistic Regression (LR), k-nearest neighbors (KNN), support vector machine (SVM), and XGBoost (XGB) (61). Given its relative simplicity and interpretability, Logistic Regression was used as the primary model reported in the main text; results for all the model types are reported as exploratory analyses in the Table 2, irrespective of their relative performance. Class weights inversely proportional to class frequency (class weight=‘ balanced ‘) parameter was used during training to account for class imbalance.

**Table 2:** PR-AUC lift comparisons made across modeling approaches by ML algorithm.

Comparison	LR (*N* = 8)	SVM (*N* = 9)	XGB (*N* = 12)	KNN (*N* = 3)
Personalized vs. chance [Well-fit]	**2.26 (1.50–2.89; *p*** **=** **.001)**	**1.93 (1.39–2.34; *p*** **=** **.004)**	**1.85 (1.43–2.11; *p*** **=** **.001)**	**2.25 (1.30–3.22; *p*** **=** **.008)**
Personalized vs. chance [Poor-fit]	1.09 (0.98–1.17; *p* = .155)	1.04 (0.95–1.15; *p* = .574)	0.95 (0.90–1.09; *p* = .938)	1.07 (1.00–1.17; *p* = .317)
Personalized vs. chance [Whole population]	**1.17 (1.05–1.25; *p*** **=** **.035)**	1.13 (1.02–1.22; *p* = .219)	1.10 (1.01–1.21; *p* = .358)	1.11 (1.01–1.20; *p* = .169)
General vs. chance [Whole population]	0.99 (0.90–1.08; *p* = .456)	0.98 (0.88–1.05; *p* = .414)	0.95 (0.87–1.03; *p* = .773)	1.04 (0.97–1.10; *p* = .573)
Personalized vs. General [Whole population]	**1.18 (*p*** **=** **.047)**	1.15 (*p* = .094)	1.14 (*p* = .119)	1.06 (*p* = .235)
Personalized vs. General [Well-fit]	**1.67 (*p*** **=** **.05)**	1.60 (*p* = .077)	**1.56 (*p*** **=** **.002)**	1.64 (*p* = .126)
Well-fit vs. Poor-fit	2.08 (1.37–2.78)	1.85 (1.30–2.29)	1.94 (1.42–2.16)	2.09 (1.20–2.98)

No *p*-value is reported for the well-fit vs. poor-fit lift ratio, as it represents the ratio between two lifts. Well-fit and poor-fit subgroups were defined from test-set lift separately for each algorithm, so subgroup comparisons are descriptive. *P*-values are unadjusted.

In the personalized models, a subset of features was selected from the training set for each participant. The top 25 features most highly correlated with the training labels were used for training and evaluation. Feature selection and the scaler used to standardize features were both fit on the training data only to avoid test-set leakage. This approach helped not only with identifying the most informative features but also with overfitting, given the relatively high number of features compared to the training samples available per participant. Alternative feature selection methods were explored, such as mutual information (62), to capture potential non-linear relationships. However, these approaches yielded less stable performance in our setting, potentially due to the sensitivity of mutual information estimation to sample size and data distribution, particularly in continuous feature spaces (63, 64).

Model performance was assessed using area under the precision recall curve (PR AUC), as this metric is appropriate for imbalanced data where there is distinct interest in correct classification of the minority class (65, 66). Because PA prevalence varied across participants, we normalized PR AUC to a lift statistic, defined as observed PR AUC divided by each participant's PA prevalence (i.e., performance relative to the base rate expected by chance). This allowed performance to be compared across participants and against a common chance baseline of 1. Given the exploratory nature of this analysis and the lack of established clinically meaningful thresholds in this field, we refrained from setting thresholds necessary for sensitivity and specificity performance metrics, and thus only PR AUC/lift were compared amongst models.

At the personalized models’ level, we categorized models into “well-fit” and “poor-fit” using the distributions of PR AUC lift across participants. Specifically, we used optimal 1-dimensional k-means clustering (k = 2) to group participants into well-fit and poor-fit. Because this grouping is derived from each participant's test-set lift, subsequent comparisons of these groups against chance, and against each other, are not independent of the grouping; we therefore report them as descriptions of observed test performance rather than as confirmatory tests. For the generalized models, we ran the training and testing using the pooled train and test sets across all the participants. We used a moving-block bootstrap (block size = 7 days) to characterize the performance of personalized models across all participants, personalized models in the well-fit group, personalized models in the poor-fit group, and generalized models. Test-set blocks were resampled with replacement and re-evaluated against each fixed, already-trained model to construct 95% confidence intervals of pooled PR AUC lift. *P*-values against chance were obtained from a separate null distribution generated within the same resampling: in each resample, the block-resampled outcome labels were paired with the fixed model's predictions for the original, non-resampled test rows. This breaks the temporal alignment between predictions and outcomes within each participant while approximately preserving each participant's outcome prevalence and autocorrelation. The *p*-value is the proportion of null lifts at least as large as the observed lift. Because the pooled lift expected under this null need not equal exactly 1 (for example, when predictions reflect between-participant differences in outcome prevalence), a confidence interval that excludes 1 can accompany a non-significant *p*-value; statements of statistical significance in this paper are based on the *p*-values. We report the PR-AUC lift for the direct comparison between personalized and generalized model performance on identical held-out sprints; we used a participant-level permutation test in which each participant's predictions were randomly reassigned, as a block, between the personalized and general model with equal probability (coinflip), generating a null distribution for the difference in lift between the two approaches. Other common evaluation metrics such as the F1-score were not considered, as they are threshold-dependent and may exhibit variability in imbalanced classification settings, particularly in heterogeneous or personalized modeling contexts (67).

#### Cross-participant exploratory analysis

We conducted an exploratory analysis to assess if the personalized models that were clustered as poor-fit reflect a genuine absence of predictable signal or are limited by the learnable patterns in their own training data. To distinguish these, we tested whether models trained on the participants clustered as well-fit could exceed chance performance on poor-fit participants’ held-out data. Successful transfer would suggest the latter — that the poor-fit group's classification could reflect limitations of an individual's own personalized model rather than a true absence of structure. Because donor models were selected using recipients’ test outcomes, this analysis is hypothesis-generating and cannot by itself establish shared predictive structure.

#### Post hoc analysis to inform future work

The *post hoc* analysis consisted of two parts. First, we assessed the degree of overlap among the top features predictive of panic attacks across participants, to characterize the level of heterogeneity in each participant's panic-attack precursor signature. For this, the Kuncheva index was used to quantify the overlap of features across personalized models. This quantity is bounded from below by −1, with maximum value of 1. A Kuncheva index of 0 indicates overlap consistent with random chance, negative values occur when feature groups have less overlap than expected by random chance, and 1 corresponds to perfect concordance (68). The pairwise Kuncheva index was computed across all possible participant pairs. This was a way to characterize the amount of heterogeneity in the predictors of PAs across individuals. To contextualize the observed mean pairwise Kuncheva index, we resampled the data from a rotation-based permutation null distribution under which correlations between features and PAs were disrupted. This provides an understanding of concordance expected due to the procedure and longitudinal data structure, beyond that expected by random chance.

Second, we performed lagged linear regression on data from two representative subjects to examine how daily deviations from an individual's mean Oura-derived behaviors across the study period relate to changes in PA risk. Changes in PA risk were determined by computing the difference in model-estimated PA likelihood between consecutive days. By regressing these daily probability changes on behavioral and physiological variables, we identified example behaviors whose day-to-day deviations were associated with increases or decreases in model-estimated PA risk.

## Results

As shown in Figure 2 (right), across all 39 participants, personalized models using the LR algorithm achieved a pooled PR-AUC lift of 1.17 (95% CI: 1.05–1.25), which was significantly higher than random chance (*p* = .035), whereas the generalized model showed a PR-AUC lift of 0.99 (95% CI: 0.90–1.08), not significantly different from random chance (*p* = .456). In the direct comparison on identical held-out sprints, personalized models outperformed the generalized model (lift ratio = 1.18, *p* = .047; Table 2). Performance varied across participants. The distribution of PR AUC lift across all participants (Figure 2 (left)) was generated by running the personalized models across all the participants (*n* = 39) using the LR algorithm, and the k-means clustering produced two different clusters, which were categorized as well-fit (*n* = 8) and poor-fit (*n* = 31) participants. By construction, the two clusters differ in lift: most of the distribution around poor-fit falls around chance (lift = 1), and the lift distribution around well-fit participants is almost double that of poor-fit.

**Figure 2:**
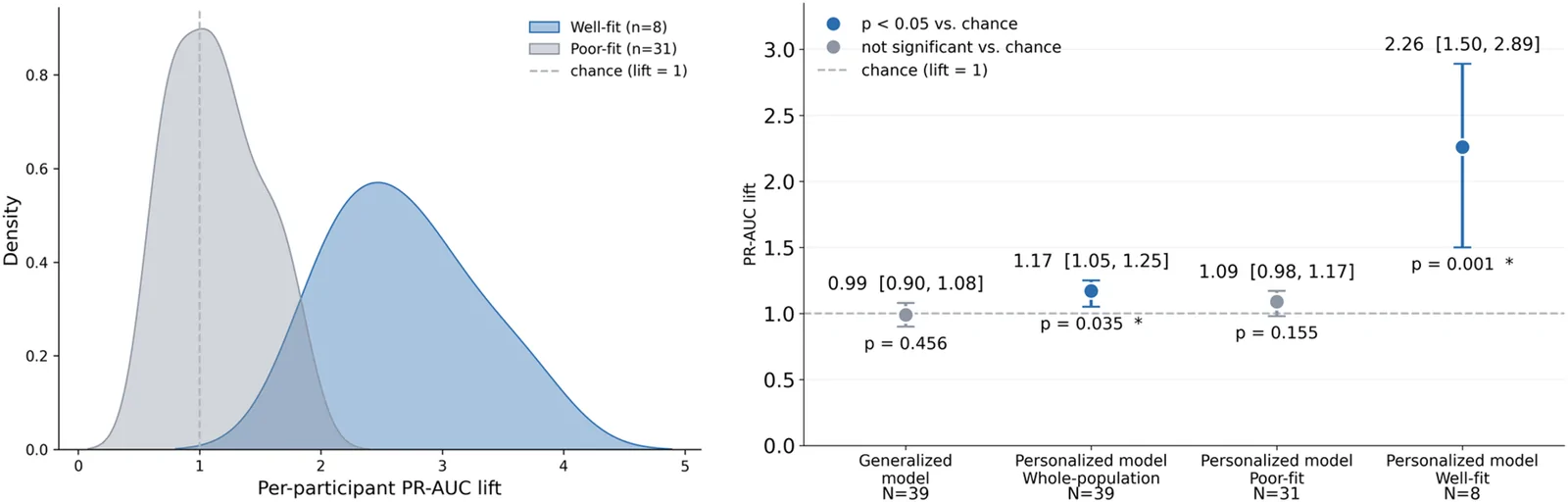
(Left) distribution of per-participant PR-AUC lift from personalized models. K-means clustering (*k* = 2) grouped participants by lift into well-fit (*n* = 8) and poor-fit (*n* = 31) subgroups; the dashed line marks chance (lift = 1). (right) PR-AUC lift relative to chance for the generalized model (*N* = 39), for personalized models across the whole population (*N* = 39), and for personalized models in the poor-fit (*N* = 31) and well-fit (*N* = 8) subgroups. Points show pooled PR-AUC lift with 95% bootstrap confidence intervals; the dashed line marks chance (lift = 1). Blue points indicate lift significantly above chance (*p* < .05); gray points indicate no significant difference from chance. Subgroups were defined from the same test-set lift, so subgroup comparisons against chance are descriptive.

The subsequent analysis in Figure 2 (right) illustrates the performance of the personalized models for the well-fit and poor-fit subgroups using the LR algorithm against chance. The well-fit personalized models (*n* = 8) showed a pooled PR-AUC lift of 2.26 (95% CI: 1.50–2.89; *p* = .001 against chance); the poor-fit personalized models showed a PR-AUC lift of 1.09 (95% CI: 0.98–1.17; *p* = .155). Because group membership was defined by the same test-set lift, the subgroup comparisons against chance are not independent tests; we report them as descriptions of observed test performance, not as evidence that well-fit participants are intrinsically more predictable. In addition to these chance-level comparisons, we directly compared well-fit against poor-fit models as relative comparisons between groups rather than against chance. Well-fit personalized models showed a greater relative advantage over chance than poor-fit models, with a lift ratio [PR-AUC lift(well-fit)/PR-AUC lift(poor-fit)] of 2.08 (95% CI: 1.37–2.78) (see Table 2 under LR). The test prevalence for well-fit and poor-fit models is M = 0.28, SD = 0.10 and M = 0.49, SD = 0.21, respectively. Personalized models trained and evaluated on the same sample using other data sources, such as stressors and DASS measures, did not differ significantly from chance (*p* > 0.05). Using stressors, the personalized models yielded a PR AUC lift of 0.90 (95% CI: 0.76–1.14); using DASS, the personalized models yielded a PR AUC lift of 1.08 (95% CI: 0.94–1.31).

Further, we implemented a persistence baseline using the count and presence of panic attacks reported within the same 7-day feature window used for the Oura-derived features, evaluated through the same train/test splits, evaluation metric, and significance-testing procedure as our primary analysis; this achieved a well-fit personalized PR-AUC lift of 1.36 (95% CI: 1.19–1.70, *p* = .0085). Adding Oura-derived features to this baseline gave a lift of 1.96 (95% CI: 1.38–2.68, *p* = .001), comparable to the Oura-only model (2.26, 95% CI: 1.50–2.89, *p* = .001). Across all 39 participants, the corresponding lifts over chance were 1.14 (95% CI: 1.06–1.29; *p* = .087) for the persistence baseline, 1.17 (95% CI: 1.05–1.25; *p* = .044) for the combined model, and 1.17 (95% CI: 1.05–1.25; *p* = .035) for the Oura-only model. The difference between the combined and persistence-only models was not formally tested, and comparing each model separately against chance is not a test of their difference. Incremental predictive value of Oura-derived features beyond recent attack history has therefore not been established in these data; the values above, including those within the well-fit subgroup, which was selected on Oura-model test performance, are reported as exploratory.

The results illustrated in Table 2 represent various comparisons made between personalized models (evaluated across the whole population and separately in well-fit and poor-fit subgroups) and generalized models (evaluated across the whole population), each tested against chance, along with between-group comparisons: personalized versus generalized models across the whole population, personalized versus generalized models within well-fit participants, and the relative lift of well-fit over poor-fit personalized models — replicated across four ML algorithms (Logistic Regression, SVM, XGBoost, and KNN; well-fit *N* = 8, 9, 12, and 3, respectively).

Across the whole population, personalized models exceeded chance for the LR algorithm (lift = 1.17, *p* = .035) but not for SVM, XGBoost, or KNN (lift = 1.13, 1.10, and 1.11; *p* = .219,.358, and.169, respectively). In the direct comparison, personalized models outperformed generalized models for the LR algorithm only (lift ratio = 1.18, *p* = .047); the ratios for SVM, XGBoost, and KNN were in the same direction but did not reach significance (1.15, 1.14, and 1.06; *p* = .094,.119, and.235, respectively), and generalized models did not differ from chance under any algorithm (lift range: 0.95–1.04, all *p* > .05; full values in Table 2). Within the subgroups, which were re-derived from test-set lift separately for each algorithm, personalized models exceeded chance for well-fit participants (lift range: 1.85–2.26, all *p* < .05) but not for poor-fit participants (lift range: 0.95–1.09, all *p* > .05); as noted above, these subgroup comparisons are descriptive. Personalized models also outperformed generalized models within well-fit participants for two algorithms, LR (lift ratio = 1.67, *p* = .05) and XGBoost (lift ratio = 1.56, *p* = .002). Across algorithms, personalized models showed an approximately twofold higher lift ratio in well-fit versus poor-fit participants [lift ratio = PR-AUC lift[well-fit]/PR-AUC lift[poor-fit]; range: 1.85–2.09; Table 2].

In the exploratory cross-participant analysis (Figure 3), some models trained on well-fit participants (donor models) performed better than random chance when applied to poor-fit (recipient) participants. In total, this yielded 8 × 31 = 248 donor–recipient comparisons. Of the 8 well-fit participants’ personalized models, 5 significantly outperformed chance for at least one of the 31 poor-fit recipients they were tested against; the remaining 3 did not significantly outperform chance for any poor-fit recipient. Of the 31 poor-fit participants, 8 had at least one well-fit donor model that significantly outperformed chance. For these same 8 participants, the pooled PR AUC lift achieved by their own personalized models — each trained and tested on that participant's own data — was M = 1.34, SD = 0.40. Substituting the best-performing significant donor model for each of these 8 participants raised the mean PR AUC lift to M = 2.10, SD = 1.33, a 56% increase. Given the exploratory nature of this analysis and the data-dependent selection of donors and recipients, these counts were not corrected for multiple comparisons. Shared predictive structure across participants has therefore not been established by this analysis; the heatmap is presented as an exploratory observation motivating independent validation.

**Figure 3:**
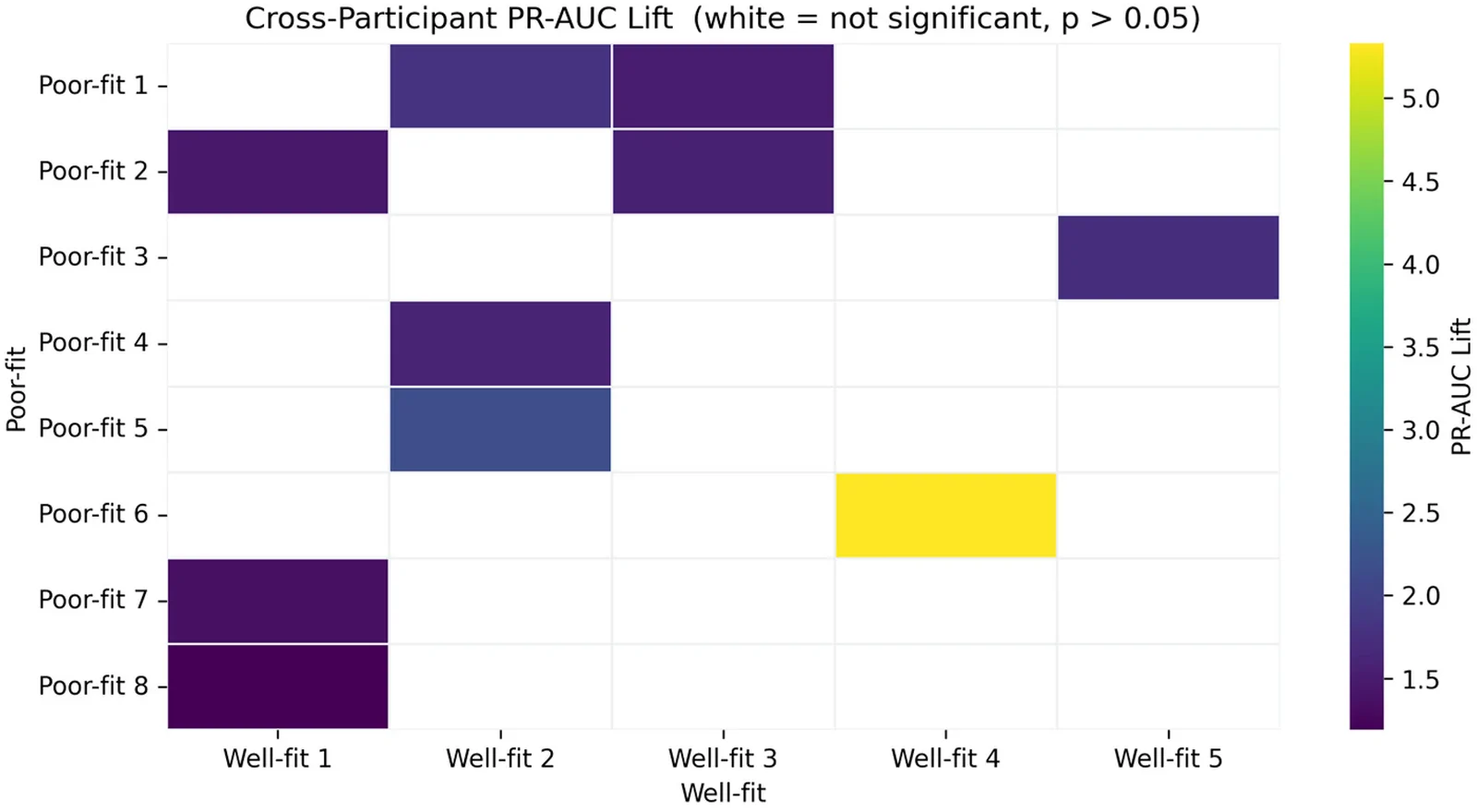
Cross-participant PR AUC lift when personalized models trained on well-fit participants are applied to poor-fit participants’ heldout test data. Columns represent well-fit (donor) participants; rows represent poor-fit (recipient) participants with at least one donor model that significantly outperformed chance (circular-shift permutation test, *p* ≤ 0.05). Cell color indicates PR AUC lift (observed PR AUC divided by the recipient’s own test-set prevalence) for each significant donor–recipient pair; white cells indicate donor models that did not significantly outperform chance for that recipient. Recipients and donors with no significant pairs are omitted. Participant identities are anonymized as Well-fit 1–5 and Poor-fit 1–8, ranked by number of significant pairs.

To showcase the utility of the personalized PA prediction models, we highlight predictions from an example participant in Figure 4. In this visualization, the participant's self-reported PA days appear in red, alongside model-predicted PA probabilities in blue (left panel- top and bottom). The participant's Oura-measured physiology and behavior are shown in black. In the top-left panel, dips in time spent in the deep sleep stage closely correspond to PA periods. The adjacent top-right boxplot reinforces this pattern (*p* < 0.01, Cohen's *d* = −0.98), showing that deep sleep time on PA days had a median value more than one standard deviation lower than on days where a PA was not experienced for this participant. The bottom-left panel shows a clear peak in breathing rate during sleep during periods when PAs were occurring, and the bottom-right boxplot confirms significantly higher breathing rate during sleep on PA days compared with days where a PA was not experienced (*p* = 0.04, Cohen's *d* = 0.51) for this participant. While the predicted probability pattern aligns well with the ground truth, there are also false positives. Experiencing a PA may require both being at increased physiological PA risk and also experiencing a triggering event.

**Figure 4:**
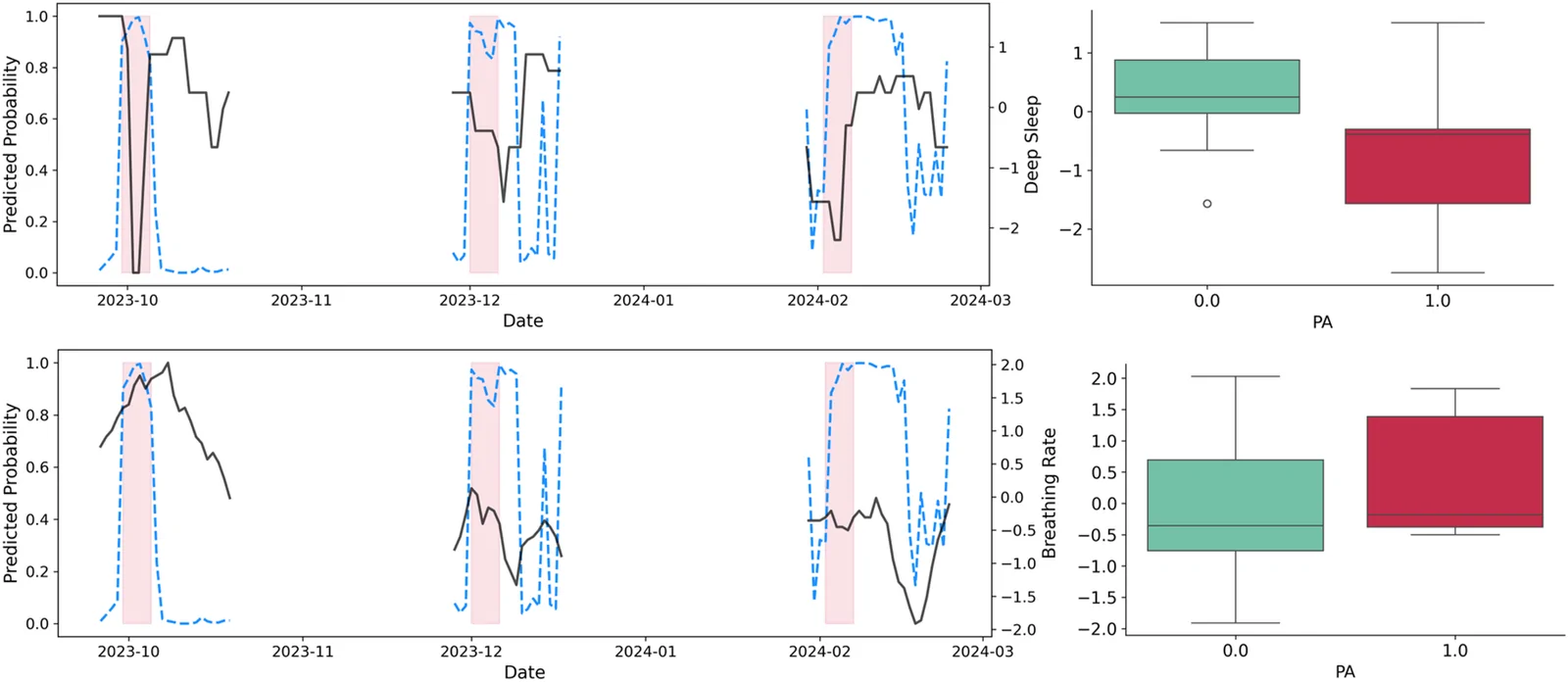
Case study of model predictions and underlying important Oura-measured features for an example participant. The left panel shows the predicted probability of experiencing a panic attack (blue, left *y*-axis), periods when panic attacks were reported (red shaded), and trends in standardized Oura measures (black, right *y*-axis) of time spent in deep sleep (top) and average breathing rate during sleep (bottom) over time. The right panel shows box plots comparing time spent in deep sleep (top) and average breathing rate during sleep (bottom) between PA (1) and non-PA days (0).

When assessing feature set overlap within the personalized models, we found an average pairwise Kuncheva index of just 0.122. Comparing this value to the permutation-based null, we found insufficient evidence to reject the hypothesis that the observed similarity arose from a combination of the selection procedure and random chance (*p* = 0.18). Further, we resampled participant data within sprints using the moving-block bootstrap (block size = 7), refitting the feature selection procedure each time to assess sensitivity of the chosen features to changes in the data within each participant. When calculating the Kuncheva index between the original features and the resamples, each participant had an average similarity index between 0.31 and 0.89, indicating moderate to strong robustness of the chosen participant feature sets.

We use lagged regressions to quantify the extent to which modeled PA risk probability increased or decreased from one day to the next with a prior change in a physiological or behavioral measurement for an example participant (Figure 5). We observed that an increase in total sleep time relative to the participant's typical total sleep time was associated with a subsequent decrease in PA probability (*β* = –0.091, R^2^ = 0.17, *p* < 0.01). Similarly, a delayed bedtime was associated with a subsequent increase in PA probability (*β* = 0.129, R^2^ = 0.18, *p* < 0.01) for another participant.

**Figure 5:**
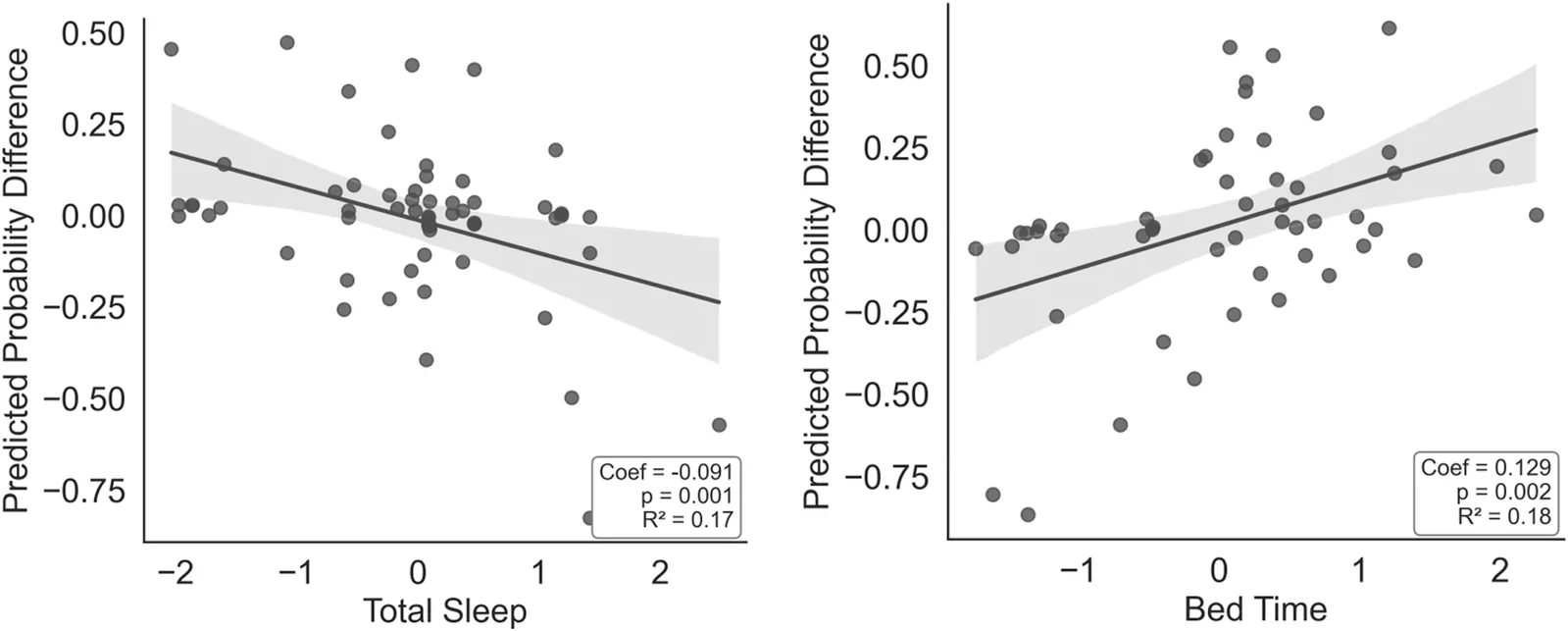
Lagged linear regressions reveal associations between day-to-day changes in the predicted probability of experiencing a PA and deviations from an example participant’s typical total sleep time (left) and time to bed (right), as measured by the Oura ring. Regression statistics (coefficient, *p*-value, and R2) are displayed in the bottom right corner of each plot.

Sensitivity analysis was carried out to assess the influence of missingness in the outcome windows. Since the outcome window is always the subsequent week following the end of the window from which the features were extracted, we allowed varying missingness levels as shown in Supplementary Figure S1. Specifically, we varied the maximum number of allowed missing outcome days from 1 to 6— ranging from a minimum of 6 observed days under the strictest criterion (maximum missing = 1 day) to a minimum of 1 observed day under the most permissive criterion (maximum missing = 6 days). Across all evaluable thresholds, the well-fit personalized model's (Logistic Regression) PR-AUC lift remained significantly above chance: lift = 1.38 [95% CI (1.05, 1.65), *p* = .012] at a maximum of 1 missing day, lift = 1.81 [95% CI (1.39, 2.32), *p* < .001] at 3 missing days, lift = 2.08 [95% CI (1.56, 2.77), *p* = .001] at 4 missing days, lift = 2.17 [95% CI (1.54, 2.75), *p* < .001] at 5 missing days, and lift = 2.26 [95% CI (1.50, 2.89), *p* = .001] at 6 missing days, with the underlying sample size ranging from 295 to 602 observations across thresholds. The lift estimate for the 2-missing-day threshold is not reported: at that threshold the well-fit subgroup comprised only 3 participants and only 120 observations, producing an unstable lift estimate and a bootstrap confidence interval that could not be reliably computed. PR-AUC lift generally increased as the missingness criterion was relaxed, stabilizing around 2.1–2.3 from 4 missing days onward, indicating that the personalized model's advantage over chance for the well-fit group was not an artifact of a specific missing-data threshold but held across a plausible range of choices, with the caveat that subgroup membership was re-derived from test-set lift at each threshold.

A further sensitivity analysis excluding feature-extraction windows that themselves contained a panic attack produced a consistent pattern: well-fit personalized models still significantly outperformed chance (lift=1.42, 95% CI: 1.23–2.26, *p* = .03), while poor-fit and generalized models did not (both *p* > .05), and well-fit models also showed a greater relative advantage over chance than poor-fit models, with a lift ratio [PR-AUC lift[well-fit]/PR-AUC lift[poor-fit]] of 1.56 (95% CI: 1.27–2.60).

## Discussion

This work investigates whether passively collected physiological and behavioral data from the Oura Ring can be used to forecast an individual's PA risk in the upcoming week and interrogates model rigor by exploring optimization and interpretability in *post-hoc* analyses. Across the complete cohort of 39 participants, personalized models using the primary algorithm (LR) achieved a modest but statistically significant lift over chance (1.17; 95% CI: 1.05–1.25; *p* = .035), whereas the generalized model did not differ from chance, and personalized models outperformed the generalized model in a direct comparison (lift ratio = 1.18, *p* = .047). The personalized modeling approach also helped in identifying promising signals of wearable-derived features associated with PA risk to inform hypothesis generation in future work. Taken together with other predictive modeling studies on panic attacks in participants with Panic Disorder (20, 36), wearable data may help inform our understanding of panic attack risk. Our findings represent an important first step toward characterizing panic attack risk transdiagnostically and suggest that personalized prediction approaches may help identify meaningful signals across a wider range of individuals than previously studied, given that most prior work has been restricted to individuals with PD.

Building generalized models trained across all the participant data, we observed modest performance (Figure 2) which was not significantly different from random chance, consistent with prior evidence demonstrating that behavioral and physiological responses to stressors differ significantly across mental health diagnoses (37–42) and even individuals (43–46). This prompted our work towards building personalized models trained separately for each participant, which modestly outperformed the generalized model across the complete cohort (lift ratio = 1.18, *p* = .047 for LR; not significant for the other three algorithms). Furthermore, while the personalized models using Oura data performed significantly better than random chance across the complete cohort for the primary algorithm (LR), the personalized models built using daily stressor count or biweekly symptoms did not differ from random chance, which suggests the potential of passively collected behavioral and physiological data in predicting PAs.

A similar well-fit/poor-fit pattern was observed under four structurally different algorithms (Table 2). Because group membership was derived from test-set lift separately for each algorithm, this consistency describes observed test performance and does not independently establish that the distinction reflects a stable characteristic of individual participants. Descriptively, personalized models exceeded chance among well-fit participants for every algorithm tested (lift range: 1.85–2.26), yet failed to do so for poor-fit participants or for generalized models applied across the whole population under any algorithm — a pattern that held whether the underlying model was linear (Logistic Regression), kernel-based (SVM), a boosted tree ensemble (XGBoost), or instance-based (KNN). Well-fit participants showed an approximately twofold higher lift ratio relative to poor-fit participants across all four algorithms (range: 1.85–2.09), indicating that this descriptive pattern was not specific to a particular modeling choice. Together, these results raise the hypothesis that panic-attack predictability may not be uniform across this cohort. This exploratory pattern is consistent with, but does not establish, heterogeneity in predictability; testing it will require defining subgroups independently of the data used to evaluate them, for example from training data or in a separate cohort. Well-fit group membership was determined independently for each algorithm, and subgroup sizes varied substantially (*N* = 3 for KNN to *N* = 12 for XGBoost); the resulting wider confidence intervals for smaller subgroups, particularly KNN, warrant cautious interpretation of those algorithm-specific estimates. *P*-values reported in Table 2 are unadjusted for multiple comparisons, consistent with the exploratory framing of this analysis, and should be interpreted as descriptive rather than confirmatory. Further, we observed a low Kuncheva index of 0.122 for the overlap of selected features across the personalized models, a value indistinguishable from agreement expected by random chance according to the permutation-based null (*p* = .18). The low feature overlap is therefore reported descriptively and does not provide evidence for person-specific predictive structure.

The well-fit subgroup's lift was also similar across choices of how outcome-window missingness was handled: PR-AUC lift remained above chance across every evaluable threshold (range: 1.38–2.26, all *p* ≤ .012), generally strengthening as the criterion was relaxed and the sample grew (Supplementary Figure S1). Because well-fit/poor-fit membership was re-derived independently at each threshold, subgroup size and composition varied across the sweep (*N* = 8, 3, 12, 9, 9, 8); overlap in membership was substantial among thresholds with adequate sample size (3–6 missing days permitted: Jaccard 0.43–0.80) but limited at the smallest threshold (2 missing days, *N* = 3), which also produced an unstable estimate (95% CI not computable). The effect also held when feature-extraction windows containing a panic attack were excluded (well-fit lift=1.42, *p* = .03; poor-fit and generalized models not significant). These analyses do not establish that Oura-derived features add predictive value beyond recent attack history: the difference between the combined and persistence-only models was not formally tested, and the recency of a panic attack contributes to predictability. Since the maximum attainable PR-AUC lift depends on test prevalence, and the well-fit and poor-fit groups differed in mean test prevalence (0.28 vs. 0.49), this should be considered when making between-group comparisons.

The case study in Figure 4 illustrates interpretable trends in deep sleep time and breathing rate during sleep that align with PA periods. Although the false-positive rate remains nontrivial, these visualizations illustrate, for a single participant, how physiological and behavioral signals may relate to panic episodes and highlight potential targets and timing for delivering interventions aimed at preventing PAs (e.g., 15, 27). As evidenced by the associations between day-to-day behavioral changes and the fluctuations in predicted risk for PA (Figure 5), models may point to specific, modifiable factors that should be investigated further in hypothesis-driven modeling. Our findings suggest plausibility between signals found to be promising links to panic risk, such as total sleep time or shifting bedtime earlier, which is targeted in well-established interventions used to reduce panic [e.g., CBT-I (69, 70), sleep hygiene (71), stimulus control techniques (72, 73). It should be noted that these lagged regressions relate the model's predicted output to its own input variables and are therefore better understood as a mechanistic interpretation of the fitted model's behavior rather than independent evidence that modifying these behaviors would alter PA risk; these findings should be viewed as hypothesis-generating rather than confirmatory.

While personalized models did not exceed chance-level performance for some participants (poor-fit), an exploratory cross-participant analysis (Figure 3) found that some models trained on other (well-fit) participants achieved better-than-chance performance on some of these participants’ held-out data. One possible interpretation, to be tested in independent data, is that poor-fit classifications reflect limitations of an individual's own personalized model rather than a true absence of predictable structure. We emphasize this finding is hypothesis-generating rather than confirmatory: model selection for each poor-fit participant relied on that participant's own test labels, and given the exploratory nature of this analysis, the many pairwise comparisons underlying Figure 3 were not corrected for multiplicity. Shared predictive structure across participants has therefore not been established by this analysis.

More hypothesis-driven research is required before this approach can inform clinical implementation. Ultimately, however, this analytic approach could support remote interventions that recommend timely risk-reduction strategies (74, 75). Realizing this potential will require an alerting system that is accurate in both directions: it must avoid false alarms (signaling risk when no attack occurs), which are known to cause user fatigue (76) and may cause psychological harm (77), and it must avoid missed attacks (failing to signal risk before an attack that does occur). It will also require that any resulting intervention be low-burden enough to sustain long-term engagement (78), and be co-designed with stakeholders who have lived experience of panic attacks to improve its usability (79, 80). Wearable devices are also an important consideration in these designs; heterogeneity in signal validation would impact prediction accuracy, and device design would impact user adherence rates and thus feasibility and clinical utility (81).

Although our results provide promising signals to inform future research on PA prediction, there are important limitations to consider. For example, our sample size is too small to compare model performance by mental health condition or demographic factors (i.e., age, race, geographic region); the cohort was relatively homogeneous despite high rates of mental health impairment; and the self-reports of panic attacks were based on the participant's interpretation. Our cross-participant analysis does not establish shared phenotypes of PAs: model selection relied on each participant's own test outcomes rather than a rule usable in advance, and any such structure would need to be validated in independent data. Similarly, the well-fit and poor-fit groups were defined from test-set performance, so whether they reflect stable participant characteristics remains to be validated, and incremental predictive value of Oura-derived features beyond recent attack history was not established. Since data were collected using a single commercially available device, future studies should include larger, more diverse populations and integrate multiple devices to develop more generalizable and device-agnostic models. This work shows a promising direction for personalized modeling approaches for predicting panic attacks and findings to inform hypothesis generation for future studies.

## Conclusion

This work presents a personalized machine learning approach for predicting PA risk using longitudinal behavioral and physiological data captured with the Oura Ring. Our analyses highlight the potential of personalized modeling approaches for investigating PA risk prediction.

## Data Availability

The datasets presented in this article are not readily available because of the sensitive nature of the mental health and wearable data collected from human participants and the terms of the informed consent under which they were obtained. Requests to access the datasets should be directed to Ryan S. McGinnis, Ryan.Mcginnis@advocatehealth.org, and are subject to approval by the University of Vermont Institutional Review Board.

## References

[1] Meuret AE White KS Ritz T Roth WT Hofmann SG Brown TA. Panic attack symptom dimensions and their relationship to illness characteristics in panic disorder. J Psychiatr Res. (2006) 40:520–7. 10.1016/j.jpsychires.2005.09.006 16293263

[2] Kessler RC Chiu WT Jin R Ruscio AM Shear K Walters EE. The epidemiology of panic attacks, panic disorder, and agoraphobia in the national comorbidity survey replication. Arch Gen Psychiatry. (2006) 63:415–24. 10.1001/archpsyc.63.4.415 16585471 PMC1958997

[3] Feldman JM Lehrer PM Borson S Hallstrand TS Siddique MI. Health care use and quality of life among patients with asthma and panic disorder. J Asthma. (2005) 42:179–84. 10.1081/JAS-54633 15962874 PMC1847650

[4] Lee Park Y Kim W Chae J-H Seob Oh K Frick KD Woo J-M. Impairment of work productivity in panic disorder patients. J Affect Disord. (2014) 157:60–5. 10.1016/j.jad.2013.12.021 24581829

[5] Tully PJ Turnbull DA Beltrame J Horowitz J Cosh S Baumeister H. Panic disorder and incident coronary heart disease: a systematic review and meta-regression in 1 131 612 persons and 58 111 cardiac events. Psychol Med. (2015) 45:2909–20. 10.1017/S0033291715000963 26027689

[6] Tietbohl-Santos B Chiamenti P Librenza-Garcia D Cassidy R Zimerman A Manfro GG. Risk factors for suicidality in patients with panic disorder: a systematic review and meta-analysis. Neurosci Biobehav Rev. (2019) 105:34–8. 10.1016/j.neubiorev.2019.07.022 31376408

[7] de Jonge P Roest AM Lim CCW Florescu SE Bromet EJ Stein DJ. Cross-national epidemiology of panic disorder and panic attacks in the world mental health surveys. Depress Anxiety. (2016) 33:1155–77. 10.1002/da.22572 27775828 PMC5143159

[8] American Psychiatric Association. Diagnostic and Statistical Manual of Mental Disorders | Psychiatry Online. Arlington, VA: DSM Library. (2013). Available online at: https://psychiatryonline.org/doi/book/10.1176/appi.books.9780890425596 (Accessed October 22, 2026).

[9] Craske MG Kircanski K Epstein A Wittchen H-U Pine DS Lewis-Fernández R. Panic disorder: a review of DSM-IV panic disorder and proposals for DSM-V. Depress Anxiety. (2010) 27:93–112. 10.1002/da.20654 20099270

[10] Goodwin RD Roy-Byrne P. Panic and suicidal ideation and suicide attempts: results from the national comorbidity survey. Depress Anxiety. (2006) 23:124–32. 10.1002/da.20151 16502406

[11] Katz C Yaseen ZS Mojtabai R Cohen LJ Galynker II. Panic as an independent risk factor for suicide attempt in depressive illness: findings from the national epidemiological survey on alcohol and related conditions (NESARC). J Clin Psychiatry. (2011) 72:1628–35. 10.4088/JCP.10m06186blu 21457675

[12] Fawcett J. Time-related predictors of suicide in major affective disorder. Am J Psychiatry. (1990) 147:1189–94. 10.1176/ajp.147.9.1189 2104515

[13] Herhaus B Siepmann M Kahaly GJ Conrad R Petrowski K. Effect of a biofeedback intervention on heart rate variability in individuals with panic disorder: a randomized controlled trial. Psychosom Med. (2022) 84:199–209. 10.1097/PSY.0000000000001031 34654028

[14] Cortez-Vázquez G Adriaanse M Burchell GL Ostelo R Panayiotou G Vlemincx E. Virtual reality breathing interventions for mental health: a systematic review and meta-analysis of randomized controlled trials. Appl Psychophysiol Biofeedback. (2024) 49:1–21. 10.1007/s10484-023-09611-4 PMC10869395 38236355

[15] McGinnis RS McGinnis EW Petrillo C Ferri J Scism J Price M. Validation of smartphone based heart rate tracking for remote treatment of panic attacks. IEEE J Biomed Health Inform. (2021) 25:656–62. 10.1109/JBHI.2020.3001573 32750933

[16] McGinnis EW Loftness B Lunna S Berman I Bagdon S Lewis G. Expecting the unexpected: predicting panic attacks from mood, Twitter, and apple watch data. IEEE Open J Eng Med Biol. (2024) 5:14–20. 10.1109/OJEMB.2024.3354208 38445244 PMC10914138

[17] McGinnis RS McGinnis EW Petrillo CJ Price M. Mobile biofeedback therapy for the treatment of panic attacks: a pilot feasibility study. In: 2019 IEEE 16th International Conference on Wearable and Implantable Body Sensor Networks (BSN) (2019), 1–4. 10.1109/BSN.2019.8771040

[18] Nahum-Shani I Smith SN Spring BJ Collins LM Witkiewitz K Tewari A. Just-in-time adaptive interventions (JITAIs) in mobile health: key components and design principles for ongoing health behavior support. Ann Behav Med. (2018) 52:446–62. 10.1007/s12160-016-9830-8 27663578 PMC5364076

[19] Kairamkonda DD McGinnis EW Price M Hidalgo JE Kim J Llorin J. Exploration of wearable sensor measures associated with panic attacks differs across mental health conditions. Front Digit Health. (2026) 8:1764371. 10.3389/fdgth.2026.1764371 42089029 PMC13136185

[20] Tsai C-H Chen P-C Liu D-S Kuo Y-Y Hsieh T-T Chiang D-L. Panic attack prediction using wearable devices and machine learning: development and cohort study. JMIR Med Inform. (2022) 10:e33063. 10.2196/33063 35166679 PMC8889475

[21] Jang S Sun T Shin S Lee H-J Shin Y-B Yeom JW. A digital phenotyping dataset for impending panic symptoms: a prospective longitudinal study. Sci Data. (2024) 11:1264. 10.1038/s41597-024-04147-6 39572578 PMC11582692

[22] Rosenfield D Zhou E Wilhelm FH Conrad A Roth WT Meuret AE. Change point analysis for longitudinal physiological data: detection of cardio-respiratory changes preceding panic attacks. Biol Psychol. (2010) 84:112–20. 10.1016/j.biopsycho.2010.01.020 20144682 PMC2978268

[23] Smits JAJ Zvolensky MJ. Emotional vulnerability as a function of physical activity among individuals with panic disorder. Depress Anxiety. (2006) 23:102–6. 10.1002/da.20146 16400629

[24] Burleson Y-AL. Relationships: panic attacks, sleeping, eating patterns, omega-3, and vitamin D. Am J Health Behav. (2025) 49:335–45. 10.5993/AJHB.49.3.7

[25] Helgadóttir B Forsell Y Ekblom Ö. Physical activity patterns of people affected by depressive and anxiety disorders as measured by accelerometers: a cross-sectional study. PLoS One. (2015) 10:e0115894. 10.1371/journal.pone.0115894 PMC4293141 25585123

[26] Caldirola D Daccò S Grassi M Alciati A Sbabo WM De Donatis D. Cardiorespiratory assessments in panic disorder facilitated by wearable devices: a systematic review and brief comparison of the wearable zephyr BioPatch with the quark-b2 stationary testing system. Brain Sci. (2023) 13:502. 10.3390/brainsci13030502 36979312 PMC10046237

[27] McGinnis E O’Leary A Gurchiek R Copeland WE McGinnis R. A digital therapeutic intervention delivering biofeedback for panic attacks (PanicMechanic): feasibility and usability study. JMIR Form Res. (2022) 6:e32982. 10.2196/32982 35113031 PMC8855306

[28] Martinez JM Garakani A Kaufmann H Aaronson CJ Gorman JM. Heart rate and blood pressure changes during autonomic nervous system challenge in panic disorder patients. Psychosom Med. (2010) 72:442–9. 10.1097/PSY.0b013e3181d972c2 20368476

[29] Gorman JM. Ventilatory physiology of patients with panic disorder. Arch Gen Psychiatry. (1988) 45:31–9. 10.1001/archpsyc.1988.01800250035006 2827599

[30] Hegel MT Ferguson RJ. Psychophysiological assessment of respiratory function in panic disorder: evidence for a hyperventilation subtype. Psychosom Med. (1997) 59:224–30. 10.1097/00006842-199705000-00003 9178332

[31] McGinnis EW Lunna S Berman I Loftness BC Bagdon S Danforth CM. Discovering digital biomarkers of panic attack risk in consumer wearables data. Annu Int Conf IEEE Eng Med Biol Soc. (2023) 2023:1–4. 10.1109/EMBC40787.2023.10339982 38083448

[32] Plehn K Peterson RA. Anxiety sensitivity as a predictor of the development of panic symptoms, panic attacks, and panic disorder: a prospective study. J Anxiety Disord. (2002) 16:455–74. 10.1016/S0887-6185(02)00129-9 12213039

[33] Grant DM Beck JG Davila J. Does anxiety sensitivity predict symptoms of panic, depression, and social anxiety? Behav Res Ther. (2007) 45:2247–55. 10.1016/j.brat.2007.02.008 17418804

[34] Hayward C Killen JD Kraemer HC Taylor CB. Predictors of panic attacks in adolescents. J Am Acad Child Adolesc Psychiatry. (2000) 39:207–14. 10.1097/00004583-200002000-00021 10673832

[35] Kessler RC Stang PE Wittchen H-U Ustun TB Roy-Burne PP Walters EE. Lifetime panic-depression comorbidity in the national comorbidity survey. Arch Gen Psychiatry. (1998) 55:801–8. 10.1001/archpsyc.55.9.801 9736006

[36] Tsai C-H Christian M Kuo Y-Y Lu CC Lai F Huang W-L. Sleep, physical activity and panic attacks: a two-year prospective cohort study using smartwatches, deep learning and an explainable artificial intelligence model. Sleep Med. (2024) 114:55–63. 10.1016/j.sleep.2023.12.013 38154150

[37] Tyson P Wilson K Crone D Brailsford R Laws K. Physical activity and mental health in a student population. J Ment Health. (2010) 19:492–9. 10.3109/09638230902968308 20812852

[38] Lewin M Lopachin J Delorme J Opendak M Sullivan RM Wilson DA. Early life trauma has lifelong consequences for sleep and behavior. Sci Rep. (2019) 9:16701. 10.1038/s41598-019-53241-y 31723235 PMC6853921

[39] Freeman D Sheaves B Waite F Harvey AG Harrison PJ. Sleep disturbance and psychiatric disorders. Lancet Psychiatry. (2020) 7:628–37. 10.1016/S2215-0366(20)30136-X 32563308

[40] Beilharz JE Paterson M Fatt S Wilson C Burton A Cvejic E. The impact of childhood trauma on psychosocial functioning and physical health in a non-clinical community sample of young adults. Aust N Z J Psychiatry. (2020) 54:185–94. 10.1177/0004867419881206 31631683

[41] Bloomfield LSP Fudolig MI Kim J Llorin J Lovato JL McGinnis EW. Predicting stress in first-year college students using sleep data from wearable devices. PLOS Digit Health. (2024) 3:e0000473. 10.1371/journal.pdig.0000473 38602898 PMC11008774

[42] Bloomfield LSP Fudolig MI Kim JN Llorin J Lovato J McGinnis EW. Predictors of anxiety trajectories in cohort of first-year college students. JAACAP Open. (2024) 3:805–16. 10.1016/j.jaacop.2024.08.004 40922789 PMC12414336

[43] Held J Vîslă A Wolfer C Messerli-Bürgy N Flückiger C. Heart rate variability change during a stressful cognitive task in individuals with anxiety and control participants. BMC Psychol. (2021) 9:44. 10.1186/s40359-021-00551-4 33731206 PMC7972344

[44] Hughes BM Howard S James JE Higgins NM. Individual differences in adaptation of cardiovascular responses to stress. Biol Psychol. (2011) 86:129–36. 10.1016/j.biopsycho.2010.03.015 20347005

[45] Teixeira AL Lee JB Nardone M Burr JF Millar PJ. Interindividual variability in renal and muscle sympathetic responses to mental stress: contributions to blood pressure regulation. J Appl Physiol (1985). (2025) 139:27–36. 10.1152/japplphysiol.00575.2024 40454654

[46] Da Estrela C MacNeil S Gouin J-P. Heart rate variability moderates the between- and within-person associations between daily stress and negative affect. Int J Psychophysiol. (2021) 162:79–85. 10.1016/j.ijpsycho.2021.02.001 33556470

[47] Leskin GA Sheikh JI. Gender Differences in Panic Disorder | Psychiatric Times. (2004) 21(1). Available online at: https://www.psychiatrictimes.com/view/gender-differences-panic-disorder (Accessed October 22, 2026).

[48] Stockdale SE Lagomasino IT Siddique J McGuire T Miranda J. Racial and ethnic disparities in detection and treatment of depression and anxiety among psychiatric and primary health care visits, 1995–2005. Med Care. (2008) 46:668–77. 10.1097/MLR.0b013e3181789496 18580385 PMC3956700

[49] Price M Hidalgo JE Bird YM Bloomfield LSP Buck C Cerutti J. A large clinical trial to improve well-being during the transition to college using wearables: the lived experiences measured using rings study. Contemp Clin Trials. (2023) 133:107338. 10.1016/j.cct.2023.107338 37722484 PMC10591842

[50] Loftness BC Hidalgo JE Cherian J Mascia G McGinnis RS McGinnis EW. Longitudinal profiles of heart rate variability in first-year college students using wearables. In: 2024 IEEE 20th International Conference on Body Sensor Networks (BSN) (2024), 1–4. 10.1109/BSN63547.2024.10780775

[51] Fudolig MI Bloomfield LSP Price M Bird YM Hidalgo JE Kim JN. Collective sleep and activity patterns of college students from wearable devices. Preprint at Available online at: 10.48550/arXiv.2412.17969 (2024).

[52] 0.01w?>Fudolig MI Bloomfield LSP Price M Bird YM Hidalgo JE Kim JN. The two fundamental shapes of sleep heart rate dynamics and their connection to mental health in college students. Digit Biomark. (2024) 8:120–31. 10.1159/000539487 PMC11250749 39015512

[53] Jetté M Sidney K Blümchen G. Metabolic equivalents (METS) in exercise testing, exercise prescription, and evaluation of functional capacity. Clin Cardiol. (1990) 13:555–65. 10.1002/clc.4960130809 2204507

[54] Henriksen A Svartdal F Grimsgaard S Hartvigsen G Hopstock LA. Polar vantage and oura physical activity and sleep trackers: validation and comparison study. JMIR Form Res. (2022) 6:e27248. 10.2196/27248 35622397 PMC9187966

[55] Miller DJ Sargent C Roach GD. A validation of six wearable devices for estimating sleep, heart rate and heart rate variability in healthy adults. Sensors. (2022) 22:6317. 10.3390/s22166317 36016077 PMC9412437

[56] Svensson T Madhawa K Nt H Chung U Svensson AK. Validity and reliability of the oura ring generation 3 (Gen3) with oura sleep staging algorithm 2.0 (OSSA 2.0) when compared to multi-night ambulatory polysomnography: a validation study of 96 participants and 421,045 epochs. Sleep Med. (2024) 115:251–63. 10.1016/j.sleep.2024.01.020 38382312

[57] Moshe I Terhorst Y Opoku Asare K Sander LB Ferreira D Baumeister H. Predicting symptoms of depression and anxiety using smartphone and wearable data. Front Psychiatry. (2021) 12:625247. 10.3389/fpsyt.2021.625247 33584388 PMC7876288

[58] Alzueta E De Zambotti M Javitz H Dulai T Albinni B Simon KC. Tracking sleep, temperature, heart rate, and daily symptoms across the menstrual cycle with the oura ring in healthy women. Int J Womens Health. (2022) 14:491–503. 10.2147/IJWH.S341917 35422659 PMC9005074

[59] Ronk FR Korman JR Hooke GR Page AC. Assessing clinical significance of treatment outcomes using the DASS-21. Psychol Assess. (2013) 25:1103–10. 10.1037/a0033100 23730826

[60] Sheehan DV Lecrubier Y Sheehan KH Amorim P Janavs J Weiller E. The Mini-International Neuropsychiatric Interview (M.I.N.I.): the development and validation of a structured diagnostic psychiatric interview for DSM-IV and ICD-10. J Clin Psychiatry. (1998) 59(Suppl 20):22–33. 9881538

[61] Pedregosa F Varoquaux G Gramfort A Michel V Thirion B Grisel O. Scikit-learn: machine learning in python. J Mach Learn Res. (2011) 12:2825–30.

[62] Peng H Long F Ding C. Feature selection based on mutual information criteria of max-dependency, max-relevance, and min-redundancy. IEEE Trans Pattern Anal Mach Intell. (2005) 27:1226–38. 10.1109/TPAMI.2005.159 16119262

[63] Kraskov A Stögbauer H Grassberger P. Estimating mutual information. Phys Rev E. (2004) 69:066138. 10.1103/PhysRevE.69.066138 15244698

[64] Vergara JR Estévez PA. A review of feature selection methods based on mutual information. Neural Comput Applic. (2014) 24:175–86. 10.1007/s00521-013-1368-0

[65] Gaudreault J-G Branco P. Empirical analysis of performance assessment for imbalanced classification. Mach Learn. (2024) 113:5533–75. 10.1007/s10994-023-06497-5

[66] Saito T Rehmsmeier M. The precision-recall plot is more informative than the ROC plot when evaluating binary classifiers on imbalanced datasets. PLoS One. (2015) 10:e0118432. 10.1371/journal.pone.0118432 25738806 PMC4349800

[67] Powers DMW. Evaluation: from precision, recall and F-measure to ROC, informedness, markedness and correlation. Preprint at Available online at: 10.48550/arXiv.2010.16061 (2020).

[68] Kuncheva LI. A stability index for feature selection. In: Proceedings of the 25th Conference on Proceedings of the 25th IASTED International Multi-Conference: Artificial Intelligence and Applications (2007), 390–5 (ACTA Press, USA).

[69] Mirchandaney R Barete R Asarnow LD. Moderators of cognitive behavioral treatment for insomnia on depression and anxiety outcomes. Curr Psychiatry Rep. (2022) 24:121–8. 10.1007/s11920-022-01326-3 35061137 PMC8948126

[70] Kanady JC Talbot LS Maguen S Straus LD Richards A Ruoff L. Cognitive behavioral therapy for insomnia reduces fear of sleep in individuals with posttraumatic stress disorder. J Clin Sleep Med. (2018) 14:1193–203. 10.5664/jcsm.7224 29991428 PMC6040781

[71] Baranwal N Yu PK Siegel NS. Sleep physiology, pathophysiology, and sleep hygiene. Prog Cardiovasc Dis. (2023) 77:59–69. 10.1016/j.pcad.2023.02.005 36841492

[72] Morin CM Bootzin RR Buysse DJ Edinger JD Espie CA Lichstein KL. Psychological and behavioral treatment of insomnia: update of the recent evidence (1998–2004). Sleep. (2006) 29:1398–414. 10.1093/sleep/29.11.1398 17162986

[73] Edinger JD Means MK. Cognitive–behavioral therapy for primary insomnia. Clin Psychol Rev. (2005) 25:539–58. 10.1016/j.cpr.2005.04.003 15951083

[74] Marwaha JS Landman AB Brat GA Dunn T Gordon WJ. Deploying digital health tools within large, complex health systems: key considerations for adoption and implementation. Npj Digit Med. (2022) 5:13. 10.1038/s41746-022-00557-1 35087160 PMC8795422

[75] Winkens LHH Naughton F Middelweerd A Perski O Simons M. Personalization of digital health interventions: challenges and current best practices. Eur Health Psychol. (2025) 24:1–4. 10.62696/ps8mbv06

[76] Shivers JP Mackowiak L Anhalt H Zisser H. Turn it off!”: diabetes device alarm fatigue considerations for the present and the future. J Diabetes Sci Technol. (2013) 7:789–94. 10.1177/193229681300700324 23759412 PMC3869147

[77] Michels EAM Gilbert S Koval I Wekenborg MK. Alarm fatigue in healthcare: a scoping review of definitions, influencing factors, and mitigation strategies. BMC Nurs. (2025) 24:664. 10.1186/s12912-025-03369-2 40542361 PMC12181921

[78] Wu A Scult MA Barnes ED Betancourt JA Falk A Gunning FM. Smartphone apps for depression and anxiety: a systematic review and meta-analysis of techniques to increase engagement. Npj Digit Med. (2021) 4:20. 10.1038/s41746-021-00386-8 33574573 PMC7878769

[79] Torous J Nicholas J Larsen ME Firth J Christensen H. Clinical review of user engagement with mental health smartphone apps: evidence, theory and improvements. Evid Based Mental Health. (2018) 21:116–9. 10.1136/eb-2018-102891 PMC10270395 29871870

[80] Blandford A Gibbs J Newhouse N Perski O Singh A Murray E. Seven lessons for interdisciplinary research on interactive digital health interventions. Digit Health. (2018) 4:2055207618770325. 10.1177/2055207618770325 29942629 PMC6016567

[81] Fuller D Colwell E Low J Orychock K Tobin MA Simango B. Reliability and validity of commercially available wearable devices for measuring steps, energy expenditure, and heart rate: systematic review. JMIR Mhealth Uhealth. (2020) 8:e18694. 10.2196/18694 32897239 PMC7509623

